# Mitochondrial Dysfunction Combined with Elevated CoQ_10_ Levels Specifically in Placental Cytotrophoblasts Suggests a Role for Mitophagy in Preeclampsia

**DOI:** 10.3390/biology15020139

**Published:** 2026-01-13

**Authors:** Jessica Ábalos-Martínez, Francisco Visiedo, María Victoria Cascajo-Almenara, Celeste Santos-Rosendo, Victoria Melero-Jiménez, Carlos Santos-Ocaña, Luis Vázquez-Fonseca, Fernando Bugatto

**Affiliations:** 1 Inflammation and Metabolic Syndrome in Pregnancy Group (CO25), Biomedical Research and Innovation Institute of Cádiz (INiBICA), 11009 Cádiz, Spain; jessica.abalos-martinez@inserm.fr (J.Á.-M.); francisco.visiedo@gm.uca.es (F.V.); celeste.santos@uca.es (C.S.-R.); victoria.melero.sspa@juntadeandalucia.es (V.M.-J.); fernando.bugatto@uca.es (F.B.); 2 Department of Human Anatomy and Embryology, University of Cádiz, 11009 Cádiz, Spain; 3 Andalusian Centre for Developmental Biology, CIBERER, National Institute of Health Carlos III (ISCIII), Pablo de Olavide University-CSIC-JA, 41013 Seville, Spain; mvcasalm@upo.es (M.V.C.-A.); csanoca@upo.es (C.S.-O.); 4 Department of Biology, University of Cádiz, 11009 Cádiz, Spain; 5 Division of Maternal-Fetal Medicine, Obstetrics and Gynecology Department, Puerta del Mar University Hospital, 11009 Cádiz, Spain; 6 Area of Obstetrics and Gynaecology, Department of Child and Mother Health and Radiology, School of Medicine, University of Cádiz, 11009 Cádiz, Spain; 7 Area of Biochemistry and Molecular Biology, Department of Biomedicine, Biotechnology and Public Health, University of Cádiz, 11001 Cádiz, Spain

**Keywords:** preeclampsia, mitochondria, cytotrophoblast, mitophagy, coenzyme Q_10_

## Abstract

Preeclampsia is a serious pregnancy disorder of unknown etiology. One of its cellular hallmarks is increased mitochondrial dysfunction in placental tissue. Further investigation into this aspect may help elucidate the molecular basis of preeclampsia. A total of 24 pregnant women who delivered by cesarean section participated in the study: *n* = 13 controls and *n* = 11 diagnosed with preeclampsia. Maternal blood samples were collected to assess the biochemical profile, and demographic and clinical data were recorded. Placental trophoblast samples were processed to isolate mitochondria and perform molecular biology assays. Women with preeclampsia exhibited the characteristic clinical features of the disease, along with biochemical alterations consistent with an inflammatory process. A significant decrease (73%) in mitochondrial DNA (mtDNA) copy number in trophoblastic tissue and a reduction in citrate synthase (CS) activity (−51%) in cytotrophoblast mitochondria-enriched fractions were observed in preeclampsia, indicating mitochondrial dysfunction accompanied by a loss of functional mitochondrial mass. In addition, we detected a marked decrease in MnSOD levels (−32%), together with an increase in the LC3II/LC3I ratio (47%) in cytotrophoblast mitochondria-enriched fractions, supporting the presence of mitochondrial alterations and suggesting the possible activation of mitophagy specifically in this cell type. Moreover, coenzyme Q_10_ (CoQ_10_) levels were elevated by 31% in trophoblastic villi. A pronounced 2.5-fold increase in CoQ_10_ normalized to CS activity (CoQ_10_/CS) was detected specifically in cytotrophoblasts from preeclamptic placentas. Importantly, we did not observe these alterations in the syncytiotrophoblast. In conclusion, preeclampsia is associated with mitochondrial dysfunction and increased CoQ_10_ levels normalized to CS activity, specifically in cytotrophoblast mitochondria, with findings being consistent with a possible involvement of mitophagy in this cell type. These findings suggest that cytotrophoblast mitochondrial metabolism may be more affected in preeclampsia compared with syncytiotrophoblasts, and that CoQ_10_ accumulation together with the possible activation of mitophagy may represent cellular defense mechanisms. Due to the limitations of the study, it should be considered exploratory and hypothesis-generating, and its results should be regarded as preliminary.

## Simple Summary

Preeclampsia is a severe pregnancy disorder associated with significant morbidity and mortality for both the mother and the baby. The causes underlying the development of preeclampsia remain unknown, but it is well established that the placenta is the organ that triggers the disease and that its mitochondrial metabolism is compromised. This study suggests that the cytotrophoblast is a placental cell type that is particularly affected at the mitochondrial level in preeclampsia. This mitochondrial dysfunction observed in this cell type is accompanied by an increase in coenzyme Q_10_ levels, which may represent a compensatory response. The data also suggest the activation of a second potential adaptive mechanism in cytotrophoblasts in response to mitochondrial dysfunction—mitophagy. This alteration and putative compensatory response were not observed in syncytiotrophoblasts. These findings highlight the possible specific involvement of the cytotrophoblast during preeclampsia, a cell type characterized by high mitochondrial activity within the placenta. Overall, these observations support the relevance of cytotrophoblast mitochondrial metabolism for future mechanistic studies aimed at better understanding preeclampsia.

## 1. Introduction

Preeclampsia (PE) is a pregnancy-specific disorder characterized by the onset of hypertension after 20 weeks of gestation, accompanied by at least one additional complication. These may include proteinuria, maternal organ dysfunction (such as acute kidney injury, hepatic impairment, neurological symptoms, hemolysis, or thrombocytopenia), or evidence of uteroplacental dysfunction, including fetal growth restriction (FGR) or an imbalance in angiogenic factors [1]. PE is also recognized as an inflammatory disease. Women with PE exhibit a state of chronic inflammation, and inflammatory markers such as C-reactive protein (CRP) and interleukins are frequently altered. Globally, preeclampsia (PE) affects approximately 2–8% of pregnancies and remains a leading cause of adverse maternal and fetal outcomes, including placental abruption, preterm delivery, and fetal growth restriction (FGR) [2]. To date, delivery of the fetus remains the only definitive intervention, which contributes to high rates of preterm birth and associated neonatal complications [3]. Although abnormal placentation is widely recognized as a key event in the pathogenesis of PE, the underlying mechanisms remain poorly understood due to its complex, multisystemic nature. A hallmark feature of PE is the insufficient invasion of extravillous trophoblasts (EVTs) and the inadequate remodeling of spiral arteries, resulting in reduced placental perfusion and hypoxia [4]. This ischemic state is accompanied by mitochondrial dysfunction and oxidative stress, leading to increased placental apoptosis and fetal growth restriction [5].

Mitochondria are often referred to as the “powerhouses” of the cell, as they are responsible for ATP production through oxidative phosphorylation [6]. The placenta—particularly its central functional unit, the trophoblastic villous or villous tree—exhibits a high metabolic rate due to the elevated mitochondrial content in its two main cell types: the cytotrophoblast (CTB) and the syncytiotrophoblast (STB). The STB arises from the fusion of cells in the underlying CTB layer. Notably, CTB and STB mitochondria differ in both structure and function, with STB mitochondria being 2–10 times smaller and displaying reduced respiratory capacity, membrane potential, and antioxidant activity [7]. Accumulating evidence indicates that mitochondrial dysfunction and elevated oxidative stress are consistently observed in placentas from women with PE [8]. Our group previously reported that the mitochondrial β-oxidation pathway of fatty acids is reduced in placental tissue from pregnancies complicated by PE [9]. Structural mitochondrial abnormalities—such as swelling, disrupted cristae, and even mitochondrial depletion—have also been documented in trophoblastic cells from PE placentas [10]. In PE, uteroplacental hypoxia increases oxidative stress in placental cells. Reactive oxygen species (ROS) promote mitochondrial lipid peroxidation in these cells [11]. These morphological and functional mitochondrial defects are believed to play a major role in disease progression, although the precise regulatory mechanisms and causal factors remain to be elucidated.

Evidence indicates that mitochondrial fission and fusion processes are dysregulated in PE. Mitofusin 2 (Mfn2), an outer mitochondrial membrane protein, plays a crucial role in maintaining mitochondrial fusion and network integrity. Reduced ATP levels and decreased Mfn2 gene expression have been reported in placentas from PE pregnancies [12]. Autophagy is essential for cellular homeostasis, and mitophagy represents the selective autophagic process targeting mitochondria. However, reports on mitophagy activation in PE placentas are conflicting. A decrease in BNIP3 expression, a key regulator of mitophagy, has been observed in PE placentas, accompanied by accumulation of p62. These changes were associated with reduced autophagy/mitophagy, accumulation of damaged mitochondria, and increased ROS levels [13]. Conversely, another study reported increased expression of the 63 kDa pro-mitophagy form of PINK1 and Parkin in PE placentas, suggesting potential activation of mitophagy. Transmission electron microscopy in the same study revealed evidence of mitophagy occurring specifically in placental CTB [14]. The observation of mitophagy activation by electron microscopy provides direct evidence that extends beyond the analysis of mitophagy-related gene expression. This finding strongly supports the notion that mitophagy is activated during PE, at least in CTB.

Coenzyme Q_10_ (CoQ_10_), a lipid-soluble molecule located in the inner mitochondrial membrane, plays a crucial role in the electron transport chain and ATP production via oxidative phosphorylation. Additionally, it functions as a potent antioxidant, particularly protecting against lipid peroxidation [15]. By maintaining mitochondrial bioenergetic homeostasis and reducing ROS, CoQ_10_ may help preserve mitochondrial function in the placenta. In PE, oxidative stress and mitochondrial dysfunction are key hallmarks of placental pathology, and an imbalance in CoQ_10_ may further exacerbate these alterations. The CTB contains a higher number of mitochondria that are metabolically more active than those in the STB. Therefore, we hypothesize that CoQ_10_ plays a particularly critical role in the CTB. Studies linking PE and CoQ_10_ are limited. In healthy pregnant Ecuadorian women, plasma CoQ_10_ levels have been reported to increase by 33%. However, in women with PE, CoQ_10_ levels are decreased in both umbilical cord blood (−67%) and maternal blood (−33%), in contrast to other lipids such as cholesterol. Conversely, placental tissue from women with PE shows an increase in CoQ_10_ levels (68%) [16]. A negative correlation has also been observed between CoQ_10_ levels in amniotic fluid during the second trimester and newborn weight [17]. In a clinical study involving 235 pregnant women at risk of PE, participants received either a placebo or 200 mg of CoQ_10_ daily from week 20 until delivery; 47 women developed PE, with 30 in the control group and 17 in the CoQ_10_-treated group. The study concluded that CoQ_10_ supplementation reduced the risk of developing PE in at-risk women [18]. Furthermore, in a murine model of PE induced by Nω-Nitro-l-argininemethyl ester (L-NAME) at 200 mg/kg, CoQ_10_ administration alleviated PE symptoms by improving placental mitochondrial function, thereby contributing to reductions in blood pressure and proteinuria [19]. The protective effects of CoQ_10_ may arise from enhanced mitochondrial bioenergetics and reduced oxidative stress, which in turn can improve endothelial function, increase nitric oxide bioavailability, and support renal function. These data indicate that CoQ_10_ represents a promising therapeutic approach for the management of PE.

This study aims to further investigate the relationship between placental mitochondrial function and PE, as well as the possible involvement of CoQ_10_ in the disease. To this end, we performed mitochondrial isolation from placental trophoblasts, with particular attention to distinguishing mitochondria derived from CTB and STB. Based on the experimental observations, we describe an exploratory framework in which PE is associated with oxidative stress and mitochondrial alterations, together with changes consistent with mitophagy activation and increased CoQ_10_ content per CS. Importantly, these alterations appear to occur specifically in CTB and were not observed in STB. Both mitophagy and elevated CoQ_10_ levels may represent cellular defense mechanisms. This approach provides new insights into the contribution of placental mitochondrial dysfunction to PE pathogenesis and may help guide future mechanistic studies focused on placental mitochondrial function.

## 2. Materials and Methods

### 2.1. Study Design and Participant Details

A cross-sectional analytical study was conducted involving pregnant women diagnosed with early-onset PE and women with healthy, uncomplicated singleton pregnancies (controls). Early-onset PE was selected because this subtype is more frequently associated with placental mitochondrial dysfunction and oxidative stress compared with late-onset PE. The participants were recruited at the University Hospital “Puerta del Mar” in Cádiz, in collaboration with the Department of Obstetrics and Gynecology and the Research Unit of INIBICA. Exclusion criteria for all the participants were congenital malformations or abnormal karyotype, gestational diabetes mellitus, obesity (BMI ≥ 30 kg/m^2^), multiple gestation, autoimmune diseases, maternal smoking, infertility treatments, conception by assisted reproductive technologies, or any other significant condition that could interfere with the study of mitochondrial function. Specific exclusion criteria for control pregnancies included hypertension and/or proteinuria. The study included participants undergoing scheduled cesarean section. We excluded vaginal deliveries to avoid the possible effect of labor exertion on placental mitochondrial metabolism. Control term placentas (*n* = 13) and PE-complicated placentas (*n* = 11) were collected from 24 pregnant women. PE was diagnosed based on the following criteria: healthy, normotensive women with a blood pressure recording of 140/90 mmHg on 2 occasions for at least 4 h intervals after week 20 of pregnancy and presence of proteinuria (300 mg per 24 h that was assessed by 24 h urine collections) and/or altered angiogenic ratio (sFlt-1/PlGF). All participants provided written informed consent after receiving detailed patient information sheets according to the Cádiz Research Ethics Committee (protocol code 166.22).

### 2.2. Sample Collection, Storage, and Assessment of Biochemical Parameters

Clinical and demographic data from the subjects were collected from medical records and recorded in protected databases. Placental samples were collected within the first 30 min after cesarean delivery. The placenta explants were obtained from the paracentral region of the placenta, specifically from the villous trophoblast, avoiding blood vessels. The placenta explants were washed twice in phosphate-buffered saline (PBS) until all traces of blood were removed, frozen in liquid nitrogen, and stored at −80 °C. Additionally, maternal blood was obtained on the day of the scheduled cesarean section for biochemical analysis. The measurement of biochemical variables in blood was carried out at our hospital using standardized methods routinely employed for patient blood analyses. All biological specimens were labeled and preserved to ensure sample traceability and participant anonymity.

### 2.3. Mitochondria Purification

Mitochondria-enriched fractions from CTB and STB were prepared using a modified protocol from [20]. The mitochondria of both STB and CTB could be separated by differential centrifugation due to their different sizes. In brief, 8 g of villous trophoblast was homogenized using the gentleMACS™ Octo Dissociator (Miltenyi Biotec, Aubur, CA, USA) with the “Human mito-tissue” (software version GM_V02.H21) program, in a specific buffer containing 1 mM EDTA, 10 mM Tris (pH 7.4), 250 mM sucrose, and protease inhibitors. The homogenate was then subjected to multiple rounds of sequential centrifugation to isolate the cytosol-enriched and mitochondria-enriched fractions from CTB and STB. The resulting fractions were aliquoted to be thawed only once, frozen in liquid nitrogen, and stored at −80 °C until analysis.

### 2.4. Citrate Synthase Activity Assay

Citrate synthase (CS) activity, a standard marker of mitochondrial quantity, was determined spectrophotometrically following the protocol described by Spinazzi et al. [21]. In brief, 3 µL of mitochondria-enriched fractions were added to a cuvette with 317 µL of MilliQ H_2_O, 500 µL of 200 mM Tris pH = 8 with 0.2% Triton, 100 µL of 1 mM DTNB, and 30 µL of 10 mM acetyl-CoA. After this, the absorbance was measured continuously at 412 nm after adding 50 µL of 10 mM oxalacetic acid. Enzymatic activity was calculated from the slope of the absorbance curve and normalized to total protein concentration using the following formula: Enzyme activity (nmol·min^−1^·mg^−1^) = (∆Absorbance/min × 1000)/[(extinction coefficient × volume of sample in mL) × protein concentration in mg/mL].

### 2.5. RNA Isolation, Reverse Transcription, and Quantitative PCR

Total RNA was isolated from 100 mg of placental explant using PRImeZOL™ Reagent (Canvax, Valladolid, Spain). RNA concentrations were quantified using the NanoDrop One Microvolume UV-Vis Spectrophotometer (Thermo Fisher Scientific, Waltham, MA, USA). Only 260/280 nm ratios between 1.8 and 2.0 samples were accepted. The mRNA was reverse-transcribed into cDNA using the iScript™ cDNA Synthesis Kit (Bio-Rad, Hercules, CA, USA). Quantitative PCR (qPCR) was performed on a CFX Connect Real-Time PCR Detection System (Bio-Rad, Hercules, CA, USA), using iTaq™ Universal SYBR^®^ Green Supermix (Bio-Rad, Hercules, CA, USA) and primers listed in Table A1. Relative mRNA levels were analyzed using the 2^−ΔΔCT^ method and normalized to β-actin [22].

### 2.6. DNA Isolation and Quantification of mtDNA

Total DNA was isolated from 25 mg of placental explant using Quick-DNA™ miniprep Plus kit (ZYMO Research, Irvine, CA, USA), following the manufacturer’s recommendations. Samples were incubated with proteinase K overnight at 55 °C. The mtDNA content in 50 ng of total DNA was measured by qPCR as described in 2.5. The PCR program (Bio-Rad CFX Maestro 1.1) consisted of an initial 10 min denaturation step at 95 °C followed by 40 cycles of denaturation (15 sec at 95 °C) and annealing/extension (1 min at 60 °C). The analyzed genes were the mitochondrial *12S-rRNA* gene and the nuclear *HPRT1* gene. The primers are listed in Table A1. For determining mtDNA copy number an independent standard curve was generated for each gene (*12S-rRNA* and *HPRT1*). mtDNA copy number values were expressed by the ratio *12S rRNA/HPRT1*.

### 2.7. Western Blot

Western blot assays were performed to quantify protein content in placental samples. Samples were homogenized manually and subsequently in RIPA lysis buffer (Thermo Scientific, Waltham, MA, USA). Protein concentration was determined using the BCA Protein Assay Kit (Thermo Scientific) following the manufacturer’s instructions. A total of 20 µg of protein for total extract or 15 µg for mitochondria-enriched samples was loaded onto 15% denaturing SDS-PAGE gels. Separated proteins were transferred to polyvinylidene fluoride (PVDF) membranes (Immobilon-P; Millipore, Burlington, MA, USA), which were blocked with 5% skim milk in TBS-T and incubated overnight at 4 °C with primary antibodies. Membranes were then incubated with specific secondary antibodies for 1 h at room temperature. All antibodies used in the study are listed in Table A2. Detection was performed by chemiluminescence using the WesternBright™ ECL kit (Advansta, San Jose, CA, USA), and signals were visualized with a ChemiDoc imaging system (Bio-Rad, Hercules, CA, USA). Band quantification was performed by densitometric analysis using ImageJ software (version 1.54g).

### 2.8. Quantification of Coenzyme Q_10_

CoQ_10_ level in placental tissue and mitochondria-enriched fractions was measured using a standard protocol for total CoQ extraction from cells, with specific modifications for tissue homogenization. For explant samples, tissue was manually homogenized using a glass-glass Dounce homogenizer (Sigma-Aldrich, St. Louis, MO, USA). Protein concentration was determined using the BCA assay, and the volume corresponding to 0.5 mg of protein for tissue samples or 0.2 mg for mitochondria-enriched samples was used. Next, 10 µM of an internal ubiquinone standard (CoQ_6_), 10× SDS, 300 µL of an ethanol/isopropanol mixture (95:5), and 700 µL of hexane were added. The samples were centrifuged at 1000× *g* for 5 min at 4 °C. The organic phase was evaporated in a SpeedVac™ DNA 130 concentrator (Thermo Scientific™, Waltham, MA, USA) at 40 °C. The dry residue was resuspended in a methanol/propanol (80:20) mixture. Two aliquots of 50 µL of the extract was directly injected into a HPLC system with a C18 reversed-phase column (15 cm, Phenomenex, Torrance, CA, USA), and a Coulochem III electrochemical detector (ECD) (Thermo Scientific™, Waltham, MA, USA). Final quantification was performed using an internal standard (CoQ_6_) and an external CoQ_10_ standard. CoQ_10_ levels were normalized to total protein content in whole tissue extracts, whereas in mitochondria-enriched fractions, normalization was performed using citrate synthase activity, which was predefined as the primary metric for data interpretation.

### 2.9. Statistical Analysis

All data are presented as the mean ± standard deviation (SD). Student’s *t*-test was used to compare differences between two groups, and one-way ANOVA was applied for comparisons among three or more experimental groups. Statistical analyses were performed using GraphPad Prism 8 software. A *p*-value < 0.05 was considered statistically significant.

### 2.10. Study Limitations

This study has several limitations that should be acknowledged. First, although the sample size is comparable to that of previous studies investigating placental mitochondrial metabolism in preeclampsia, some analyses were performed in smaller subsets due to the limited availability of mitochondrial material. This may reduce the generalisability of the findings.

Second, mitochondria-enriched fractions were analyzed rather than fully purified mitochondrial preparations. While cytosolic contamination was negligible, as indicated by the absence of LDHA, contamination from other organelles, such as the endoplasmic reticulum, cannot be entirely excluded.

Third, mitophagy was evaluated using static markers, and no dynamic mitophagy flux assays were performed. As a result, any conclusions regarding mitophagy are indirect and should be interpreted cautiously. Future studies employing lysosomal inhibition or other flux-based methodologies will be required to directly assess mitophagy dynamics.

In addition, protein-based normalization was applied for some mitochondrial measurements. Although this approach is commonly used, disease-associated alterations in mitochondrial mass or protein composition in preeclampsia may influence normalization accuracy.

Finally, although statistical analyses were limited to pairwise comparisons using Student’s *t*-test, multiple independent tests were conducted across different parameters, which may increase the risk of type I error.

Overall, in light of these limitations, this study should be considered exploratory and hypothesis-generating. The findings represent preliminary, cell-type-specific observations that generate testable hypotheses for future mechanistic and interventional studies in preeclampsia.

## 3. Results

### 3.1. Clinical and Biochemical Characteristics of Pregnant Women

The clinical characteristics of 24 pregnant women were analyzed, including 13 controls and 11 with PE (Table 1). No significant differences were observed in maternal age, gestational weight gain, parity, or number of previous miscarriages. In contrast, significant differences (*p* ≤ 0.05) were found in variables typically associated with PE, such as placental weight (520 vs. 303 g, *p* = 0.00008), neonatal birth weight (3205 vs. 1669 g, *p* = 0.000002), pre-pregnancy BMI (22.8 vs. 28.5 kg/m^2^, *p* = 0.004), and gestational age at delivery (38.3 vs. 32.4 weeks, *p* = 0.000006). Moreover, 64% of pregnancies in the PE group presented with fetal growth restriction (FGR), compared with none in the control group.

Blood biochemical characteristics were analyzed in 18 pregnant women, including 10 controls and 8 with PE (Table 2). Significant differences (*p* ≤ 0.05) were observed in C-reactive protein (CRP) (3.45 vs. 9.36 mg/L, *p* = 0.039) and interleukin-6 (IL-6) (20.11 vs. 34.43 pg/mL, *p* = 0.006). No significant differences were found in the remaining parameters; however, slightly higher levels of triglycerides (189.0 vs. 292.0 mg/dL, *p* = 0.068), glucose (65.14 vs. 98.5 mg/dL, *p* = 0.051), and urea (33.8 vs. 49.5 mg/dL, *p* = 0.06) were detected in the PE group.

### 3.2. Quantification and Functional Assessment of Mitochondria-Enriched Fractions from Placental Trophoblasts

**Figure 1:**
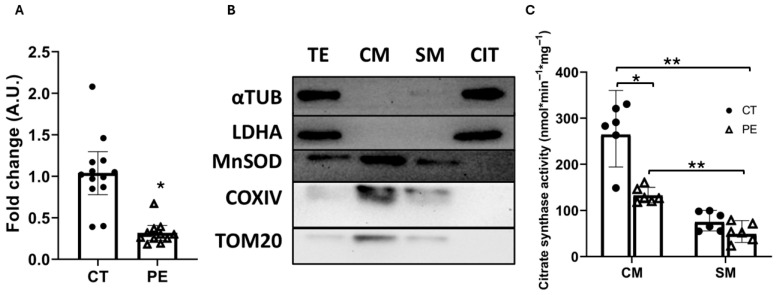
Quantification and functional assessment of mitochondria-enriched fractions from the placental trophoblast. (**A**) Quantification of mtDNA. A relative amount of mtDNA was estimated based on the measurement in total DNA by qPCR of the gene encoded by nuclear DNA (nDNA), HPRT1, and the gene encoded by mtDNA, 12S. (*n* = 13). (**B**) Control of the mitochondrial enrichment process. TE (total extract), CIT (cytosol), CM (mitochondria-enriched fraction from the cytotrophoblast), SM (mitochondria-enriched fraction from the syncytiotrophoblast). (**C**) Measurement of citrate synthase activity in the mitochondria-enriched fractions. *n* = 6. Data are presented as mean differences with 95% confidence intervals (95% CI). (*) *p* ≤ 0.05, (**) *p* ≤ 0.01.

As an initial estimate of mitochondrial mass in PE, we quantified mitochondrial DNA (mtDNA) levels in placental trophoblast tissue explants. Consistent with previously published data [23], we observed a 73% reduction in mtDNA content in PE samples (Figure 1A). However, this indirect measure of mitochondrial mass does not allow differentiation between mitochondria from CTB and STB. Therefore, we performed mitochondrial purification from trophoblastic tissue.

To accurately assess mitochondrial health in the placenta during PE, we isolated mitochondria-enriched fractions from the two most mitochondrially active trophoblastic cell types, CTB and STB [24]. We successfully obtained mitochondria-enriched fractions from CTB (CM) and STB (SM) (Figure 1B). The complete images of the original Western blots are provided in the Supplementary Material (Figure S1). In these fractions, the cytosolic soluble protein marker LDHA was undetectable, and only a faint signal for the cytoskeletal marker α-tubulin was observed. This minimal α-tubulin presence is expected, as mitochondria-associated membranes (MAMs) and parts of the cytoskeleton are often co-isolated during mitochondrial purification. It should be noted that we did not obtain fully purified mitochondrial fractions, but rather mitochondria-enriched fractions, and the possibility of cross-contamination exists. We cannot exclude the presence of contamination from other organelles, such as the endoplasmic reticulum. The mitochondrial matrix protein MnSOD served as an indicator of mitochondrial integrity: its presence in mitochondrial-enriched fractions and absence in the cytosolic fraction confirmed the preservation of intact organelles. The predominance of MnSOD in purified mitochondrial fractions, together with the lack of LDHA contamination, supports the suitability of these fractions for downstream analyses. Mitochondrial enrichment was quantified using MnSOD as a marker of structurally intact mitochondria. The analysis revealed a 3.2-fold enrichment in CM and a 2.1-fold enrichment in SM compared with TE.

Once the mitochondria-enriched fractions (CM and SM) were obtained, we enzymatically measured citrate synthase (CS) activity in these fractions (Figure 1C). CS activity is a widely accepted marker reflecting mitochondrial bioenergetic status as well as mitochondrial content and structural integrity [25]. As expected, CS activity was significantly lower in SM compared to CM in both CT and PE groups (reductions of 72% and 60%, respectively), reflecting the naturally lower mitochondrial activity and content of the syncytiotrophoblast. Moreover, we observed a marked decrease in CS activity within the CM fraction from PE samples (−51%) compared with CT, whereas no significant differences were detected in SM. These observations are consistent with a reduction in mitochondrial mass and/or bioenergetic capacity in PE samples, predominantly affecting cytotrophoblast-derived fractions, and are indicative of altered mitochondrial function in this cell type.

### 3.3. Analysis of the Autophagy and Mitophagy Process in Placental Trophoblast

**Figure 2:**
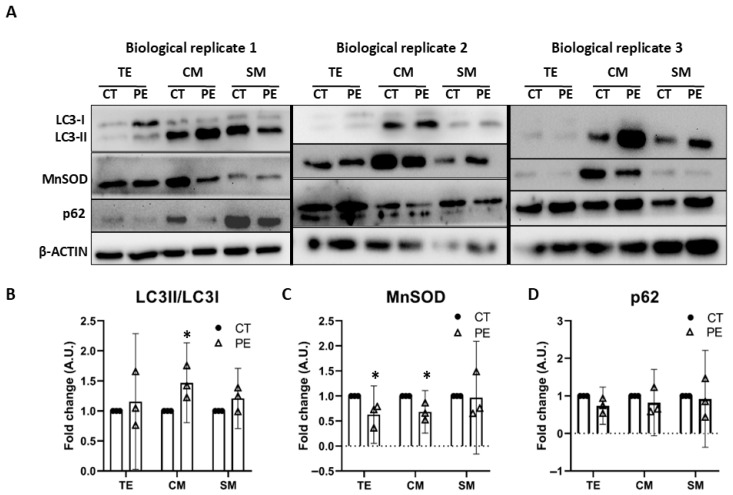
Evaluation of the mitophagy process in placental trophoblast. (**A**) Western blot analysis of autophagy markers. β-Actin was used as a loading control. Three independent biological replicates are shown (*n* = 3). (**B**) Quantification of Western blots. CM (cytotrophoblast mitochondria-enriched fraction), SM (syncytiotrophoblast mitochondria-enriched fraction) for LCII/LCI. (**C**) Quantification of Western blots for MnSOD. (**D**) Quantification of Western blots for p62. Data are presented as mean differences with 95% confidence intervals (95% CI). (*) *p* ≤ 0.05.

After observing a significant decrease in CS activity in mitochondrial fractions from CTB in PE, we sought to determine, by Western blot analysis, whether this reduction was associated with changes consistent with increased mitochondria-specific autophagy, or mitophagy. Figure 2A shows Western blots of 3 biological replicates illustrating the obtained results. The complete images of the original Western blots are provided in the Supplementary Material (Figure S1). Autophagy was quantified in total trophoblast extract (TE) using the widely accepted LC3II/LC3I ratio (Figure 2B). A slight upward trend was observed in the PE group, which may reflect altered autophagy-related signaling. When analyzing the mitochondria-enriched fractions, we detected a 47% increase in the LC3II/LC3I ratio in CM, consistent with an increased presence of structures associated with autophagic and/or mitophagic processes in this fraction. In the SM fraction, a minor upward trend was also observed, although it did not reach statistical significance.

Similarly to CS activity, to quantify mitochondrial functionality, we used the mitochondrial matrix marker MnSOD (Figure 2C). A 39% decrease in MnSOD was found in TE from PE samples. Similarly, CM showed a 32% reduction, whereas no significant changes were detected in SM. These observations are consistent with altered mitochondrial-associated parameters in CM from PE samples, whereas SM appeared comparatively unaffected.

Finally, to further investigate autophagic/mitophagic flux, we examined the p62 marker (Figure 2D). This protein serves as an indicator of autophagic flux because it participates in multiple autophagic structures (phagophores, autophagosomes, and autolysosomes) and is degraded along with its cargo [26]. The data showed considerable variability among samples. Despite this variability, we did not observe consistent p62 accumulation in PE, possibly suggesting that the autophagic flux remains functionally active in the autophagic/mitophagic process. However, although the p62 results are compatible with preserved autophagic flow, additional assays would be required to confirm this observation.

In summary, in CM from PE samples, an increase in the LC3II/LC3I ratio was observed, reflecting a higher abundance of autophagy-related structures. This was accompanied by a significant decrease in MnSOD, consistent with a reduction in the amount of functional mitochondria. Together, these findings are compatible with the presence of mitophagy-related processes in CM during PE, a pattern not observed in SM. These data support the specific reduction in mitochondrial bioenergetics and/or mitochondrial mass observed in CM, as discussed in Section 3.2. These results should be interpreted with caution, as they need to be confirmed in future mitophagy studies that include dynamic flux experiments.

### 3.4. Distribution of Coenzyme Q_10_ in Placental Trophoblast

**Figure 3:**
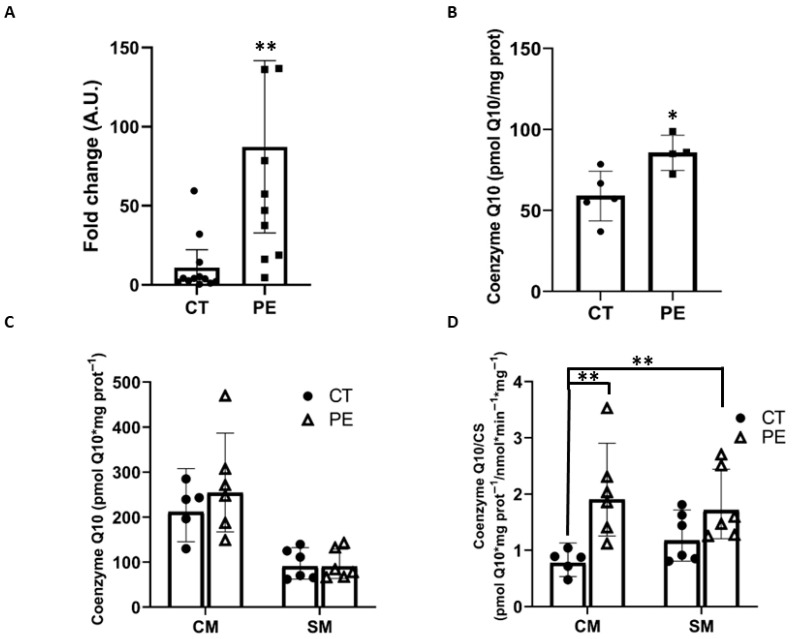
Distribution of coenzyme Q_10_ in placental trophoblast. (**A**) Quantification of mRNA for the gene involved in the coenzyme Q_10_ synthesis pathway *COQ2*. (*n* = 11) (**B**) Measurement of coenzyme Q_10_ in placental trophoblast explants (*n* = 5). (**C**) Measurement of coenzyme Q_10_ in mitochondria-enriched fractions. (*n* = 6). (**D**) Measurement of coenzyme Q_10_ in mitochondria-enriched fractions normalized by citrate synthase activity (*n* = 6). Data are presented as mean differences with 95% confidence intervals (95% CI). (*) *p* ≤ 0.05, (**) *p* ≤ 0.01.

As mentioned above, a 70% increase in CoQ_10_ levels in placental tissue during PE has been reported [16]. We measured CoQ_10_ in trophoblast villous explants and observed a more moderate increase of 31% in PE (Figure 3B). We also examined the expression of *COQ2*, a key gene in the CoQ_10_ biosynthesis pathway (Figure 3A), and found an 8-fold upregulation of its mRNA in the PE group. These findings support the observed increase in CoQ_10_ in placental trophoblast villous tissue. Next, we investigated whether this increase occurred similarly in CTB and STB by measuring CoQ_10_ in the mitochondria-enriched fractions from CM and SM (Figure 3C). Although differences were not statistically significant, we observed a 24% increase in CoQ_10_ in CM from PE samples, which is in line with the trend observed in whole trophoblastic tissue. No differences were detected in SM. However, it is important to consider the mitochondrial dysfunction accompanied by a significant reduction in the amount of functional mitochondria in CM during PE. Therefore, CoQ_10_ levels were normalized to healthy mitochondrial content. For this purpose, CS activity was used to express CoQ_10_ per functional mitochondrion. (Figure 3D). In PE, CoQ_10_ levels in CM increased significantly, by more than 2.5-fold, whereas in SM only a slight upward trend was observed. This trend resulted in statistically significant differences between CM in controls and SM in PE. Overall, these results show that CoQ_10_ levels are elevated in placental trophoblast samples from PE pregnancies, with a more pronounced increase observed in cytotrophoblast-derived mitochondrial fractions when normalized to CS activity. Representative examples of chromatograms for CoQ_10_ measurements from each sample are provided in the Supplementary Material (Figure S2).

## 4. Discussion

This study investigates the relationship between PE pathology and placental mitochondrial dysfunction. Our findings support the notion that mitochondrial impairment in the placental trophoblast plays a key role in the pathophysiology of PE, with a particularly pronounced effect on the CTB. We emphasize the importance of this cell type relative to the STB. Clinically, significant differences were observed in common PE-related variables, including neonatal weight, placental weight, and gestational age. The latter two parameters are known to be negatively associated with impaired fetal development and maturation, as reflected by the high incidence of FGR in the PE group. Additionally, pregnant women with PE exhibited a significantly higher average BMI (overweight, BMI ≥ 25), a recognized risk factor for developing PE [27]. Biochemically, we detected elevated inflammatory markers, such as CRP and IL-6, consistent with the systemic inflammatory state previously reported in PE [28]. Together, these clinical and biochemical data indicate that the placental samples analyzed reflect typical features of PE.

In terms of mitochondrial analysis, we observed a 73% reduction in mtDNA copies in PE placentas, consistent with the presence of mitochondrial dysfunction accompanied by a marked reduction in mitochondrial number. As a second marker of mitochondrial health, we measured CS activity and detected a 51% reduction in CM in PE, in agreement with the decline in mtDNA copy number. CS activity is a marker of mitochondrial mass and also reflects the bioenergetic status of mitochondria. Clinically, it is a widely used marker for the diagnosis of mitochondrial diseases, as it provides an estimate of functional mitochondrial mass. Similarly, the antioxidant enzyme MnSOD serves as an additional marker of antioxidant capacity and, consequently, of the amount of functional mitochondria. In our data, we quantified MnSOD levels by Western blot and also observed a pronounced decrease (−32%) in CTB during PE. Taken together, the mtDNA copy number, CS activity, and MnSOD content are consistent with the presence of mitochondrial dysfunction—possibly associated with a reduction in mitochondrial mass—specifically in CTB during PE. This dysfunction was not observed with the same severity in STB.

After identifying the mitochondrial dysfunction in placental CTB, we sought to determine whether this dysfunction was accompanied by variations in markers related to mitophagy. In CM, we observed a significant 47% increase in the LC3II/LC3I ratio and a 32% decrease in MnSOD levels. These findings are compatible with an activation of mitophagy in this cell type. The decrease in MnSOD levels in CTB, together with the decline in CS activity, supports the presence of mitochondrial dysfunction and a reduction in the amount of functional mitochondria. Consequently, an increase in mitophagy is expected, as suggested by the elevated LC3II/LC3I ratio in this cell type. This phenomenon appears specific to CTB and was not observed in STB. Previous studies have reported structures indicative of mitophagy in CTB from PE placentas [14], but these observations were neither quantified nor demonstrated to be cell-type specific. Nonetheless, we cannot exclude the alternative possibility that the observed LC3 lipidation occurs independently of autophagic flux. Importantly, mitophagy seems to be activated in CTB during PE—pending confirmation from further studies—and our data suggest that autophagic flux remains functional, as no accumulation of p62 was detected. We therefore propose that CTB exhibits mitochondrial dysfunction accompanied by an increased LC3II/LC3I ratio, suggesting—and consistent with previous reports—a role for mitophagy in CTB during PE.

On the other hand, we observed a significant upregulation of *COQ2*, a key gene in the CoQ_10_ biosynthesis pathway, in placentas with PE. Furthermore, CoQ_10_ levels in placental tissue were increased by 31% in PE, a more moderate elevation than previously reported. This discrepancy may be explained by differences in study populations. In the study by Terán et al., participants were women residing in Quito (2800 m above sea level), all under 25 years of age, nulliparous, and with unspecified placental sampling regions. In contrast, our cohort included women living in Cádiz (at sea level), with a mean age of 33.3 years, including both nulliparous and multiparous participants, and placental trophoblast tissue was consistently sampled. When analyzing CoQ_10_ levels in mitochondrial fractions, no statistically significant differences were observed; however, there was a slight tendency for CoQ_10_ to increase in CTB in PE. Importantly, the observed mitochondrial dysfunction in CTB during PE must be taken into account when evaluating CoQ_10_ levels in these fractions. As previously discussed, the data on mtDNA copy number, CS activity, and MnSOD levels indicate a reduction in functional mitochondrial mass in CTB. Under these conditions, a proportional decrease in CoQ_10_ content in CTB would be expected; however, such a reduction was not observed. This finding is compatible with the existence of a compensatory response in which CTB attempts to counterbalance mitochondrial dysfunction by increasing CoQ_10_ levels. It is plausible that this reflects a concentration of CoQ_10_ within the remaining healthy mitochondria. Therefore, we propose that CoQ_10_ levels should be normalized to functional mitochondrial mass, represented by CS activity. Interestingly, in PE, we observed a 2.5-fold increase in CoQ_10_ when normalized to functional mitochondrial mass in CM, further supporting the idea of a compensatory mitochondrial response. CoQ_10_ is a component of the electron transport chain (ETC) and also functions as an antioxidant against lipid peroxidation. The increase in placental lipid peroxidation in PE could represent another factor within the resulting mitochondrial dysfunction, potentially inducing an upregulation of CoQ_10_ biosynthesis as a protective response. We observed that mitochondrial dysfunction specifically affects the CTB. Accordingly, CTB may exhibit a more robust compensatory response, as mitochondrial homeostasis is more severely compromised in this cell type, resulting in a greater increase in CoQ_10_ levels when normalized to CS activity compared with STB.

Overall, these results support an association between PE and significant alterations in mitochondrial metabolism in CTB, leading to a reduction in the amount of functional mitochondria and suggesting a possible activation of mitophagy. This process could mediate the selective preservation of healthy mitochondria. In parallel, we observed an increase in CoQ_10_ concentration, possibly within this population of healthy mitochondria, as a compensatory response to the mitochondrial dysfunction. These effects were not observed in STB, likely due to its distinct structural and metabolic characteristics, such as smaller mitochondria, lower membrane potential, and reduced antioxidant capacity. We propose that both the potential activation of mitophagy and the increase in CoQ_10_ may act as cellular defense mechanisms against mitochondrial dysfunction in CTB during PE. Future studies employing mitophagy modulators or CoQ_10_ supplementation could provide new therapeutic strategies to mitigate the progression of PE.

## 5. Conclusions

Placental mitochondrial dysfunction is widely recognized as a hallmark of PE. However, in this study, we observed a marked mitochondrial dysfunction specifically in CTB during PE. This mitochondrial impairment was accompanied by an increase in CoQ_10_ levels within CTB mitochondria, which may reflect an adaptive response to the dysfunction. The elevation of the LC3II/LC3I ratio, together with the decrease in markers of functional mitochondrial mass, is consistent with changes in mitophagy-related markers specifically in CTB during PE. These observations indicate that a possible activation of mitophagy and elevated CoQ_10_ levels in CTB may represent adaptive responses aimed at counteracting mitochondrial dysfunction and the excessive ROS production characteristic of PE. These conclusions are presented as preliminary and hypothesis-generating and should therefore be interpreted with appropriate caution. Further studies will be required to validate our findings and to clarify their mechanistic relevance for placental mitochondrial biology in PE.

## Figures and Tables

**Table 1:** Clinical characteristics of recruited participants.

Variable	Control (*n* = 13)	PE (*n* = 11)	*p* Value
Maternal age (years)	33.5 ± 6.9	33.0 ± 6.0	0.84
Pre-pregnancy maternal BMI (kg/m^2^)	22.8 ± 3.1	28.5 ± 5.3	0.004 **
Pregnancy weight gain (kg)	13.4 ± 7.8	11.4 ± 5.2	0.522
Gestational age (weeks)	38.3 ± 1.1	32.4 ± 2.9	0.0000006 **
Nulliparity (%)	45.5%	54.5%	0.35
Previous abortions number	0.77 ± 1.23	0.40 ± 0.70	0.408
Placental weight (g)	520 ± 67	303 ± 800	0.00008 **
Neonatal birth weight (g)	3205 ± 303	1669 ± 800	0.000002 **
FGR (%)	0	64	<0.001 **
Neonatal sex (female, %)	38	55	0.35

Data expressed as means ± SD. BMI, body mass index. FGR, fetal growth restriction. (**) *p* ≤ 0.01.

**Table 2:** Biochemical characteristics of recruited participants.

Variable	Control (*n* = 10)	PE (*n* = 8)	*p* Value
Triglycerides (mg/dL)	189.0 ± 129.5	292.0 ± 78.0	0.068
Cholesterol (mg/dL)	198.2 ± 105.0	250.9 ± 89.2	0.20
LDL Cholesterol (mg/dL)	116.8 ± 73.4	126.9 ± 79.2	0.78
HDL Cholesterol (mg/dL)	64.2 ± 25.4	70.4 ± 30.6	0.65
Aspartate aminotransferase (AST) (U/L)	24.2 ± 8.6	34.12 ± 40.5	0.46
Alanine transaminase (ALT) (U/L)	16.8 ± 9.8	15.4 ± 4.4	0.70
Glucose (mg/dL)	65.14 ± 12.1	98.5 ± 48.0	0.051
Insulin (μUl/mL)	16.14 ± 9.0	9.44 ± 8.0	0.12
Urea (mg/dL)	33.8 ± 17.2	49.5 ± 16.7	0.06
Creatinine (mg/dL)	0.66 ± 0.25	0.77 ± 0.25	0.34
C-reactive protein (CRP) (mg/L)	3.45 ± 4.46	9.36 ± 6.68	0.039 *
Interleukin 6 (IL-6) (pg/mL)	20.11 ± 4.0	34.43 ± 13.7	0.006 **

Data expressed as means ± SD. (*) *p* ≤ 0.05, (**) *p* ≤ 0.01.

## Data Availability

The original contributions presented in this study are included in the article/Supplementary Material. Further inquiries can be directed to the corresponding author.

## Supplementary Materials

The following supporting information can be downloaded at https://www.mdpi.com/article/10.3390/biology15020139/s1, Figure S1: The complete images of the original Western blots. Figure S2: CoQ Chromatograms.

## Abbreviations

The following abbreviations are used in this manuscript:

PE	Preeclampsia
FGR	Fetal growth restriction
ATP	Adenosine triphosphate
CTB	Cytotrophoblast
STB	Syncytiotrophoblast
ROS	Reactive oxygen species
Mfn2	Mitofusin 2
BNIP3	*Bcl-2 interacting protein 3*
PINK1	PTEN-induced kinase 1
CoQ_10_	Coenzyme Q_10_
L-NAME	Nω-Nitro-l-argininemethyl ester
BMI	*Body mass index*
CS	*Citrate synthase*
DTNB	5,5′-dithiobis-(2-nitrobenzoic acid)
mtDNA	*Mitochondrial DNA*
nDNA	*Nuclear DNA*
cDNA	*Complementary DNA*
CRP	*C-reactive protein*
IL-6	*Interleukin 6*
MAMs	mitochondria-associated membranes
LDHA	Lactate dehydrogenase A
MnSOD	Manganese Superoxide Dismutase
LC3	Microtubule-associated protein 1A/1B-light chain 3
TE	Total extract

## Appendix A

### Appendix A.1

**Table A1:** Primer sequences used for qPCR analysis.

Gen	Primer	Sequence (5′→3′)
*HPRT1*	Foward	ACGTCTTGCTCGAGATGTGA
	Reverse	GAGCACACAGAGGGCTACAA
*12S*	Foward	CCACGGGAAACAGCAGTGAT
	Reverse	CTATTGACTTGGGTTAATCGTGTGA
*β* *-Actin*	Foward	CGTACCACTGGCATCGTGAT
	Reverse	GTGTTGGCGTACAGGTCTTTG
*COQ2*	Foward	CTATTCTGATGCGTGGAG
	Reverse	CGATTGGCTGTTCTTGTA
*COQ4*	Foward	CTGTCCTCCGTCGGCTCTG
	Reverse	GCGGTGTCCTGTGGTCTCC
*COQ7*	Foward	AGTTCTGATGCCCTTGTG
	Reverse	GTTGTAGTGATGTGCTATGC

HPRT1 Hypoxanthine-guanine phosphoribosyltransferase 1, 12S Mitochondrially encoded 12S ribosomal RNA.

### Appendix A.2

**Table A2:** Primary antibodies used for Western blot.

Target	Host	Supplier (Catalog Nº)	Predicted Size (KDa)	Dilution
LC3A/B	Rabbit	Cell Signaling (Danvers, MA, USA) (D3U4C)	16/14	1:1000
LDHA	Rabbit	Cell Signaling (Danvers, MA, USA) 3582T (C4B5)	37	1:1000
COXIV	Rabbit	Cell Signaling (Danvers, MA, USA) 4850T (3E11)	17	1:1000
P62	Rabbit	Abclonal (Woburn, MA, USA) A11250	62	1:1000
β-Actin	Mouse	Abcam (Cambridge, UK) ab6276 (AC-15)	42	1:1000
MnSOD	Rabbit	Sigma-Aldrich (St. Louis, MO, USA) 06-984	24	1:1000
α-Tubulin	Mouse	Sigma-Aldrich (St. Louis, MO, USA) (T5168)	50	1:10,000
TOM20	Rabbit	Proteintech (Planegg-Martinsried, Germany) 11802-1-AP	16	1:1000

LC3 microtubule-associated protein 1 light chain 3, LDHA lactate dehydrogenase A, COXIV cytochrome c oxidase IV, MnSOD manganese superoxide dismutase-2, TOM 20 mitochondrial import receptor.
