# Supplementary material for: Mitochondrial Dysfunction Combined with Elevated CoQ10 Levels Specifically in Placental Cytotrophoblasts Suggests a Role for Mitophagy in Preeclampsia
Source: Biology (Basel). 2026 Jan 13;15(2):139. doi: 10.3390/biology15020139 (PMC12838238; doi:10.3390/biology15020139)

Figure S1

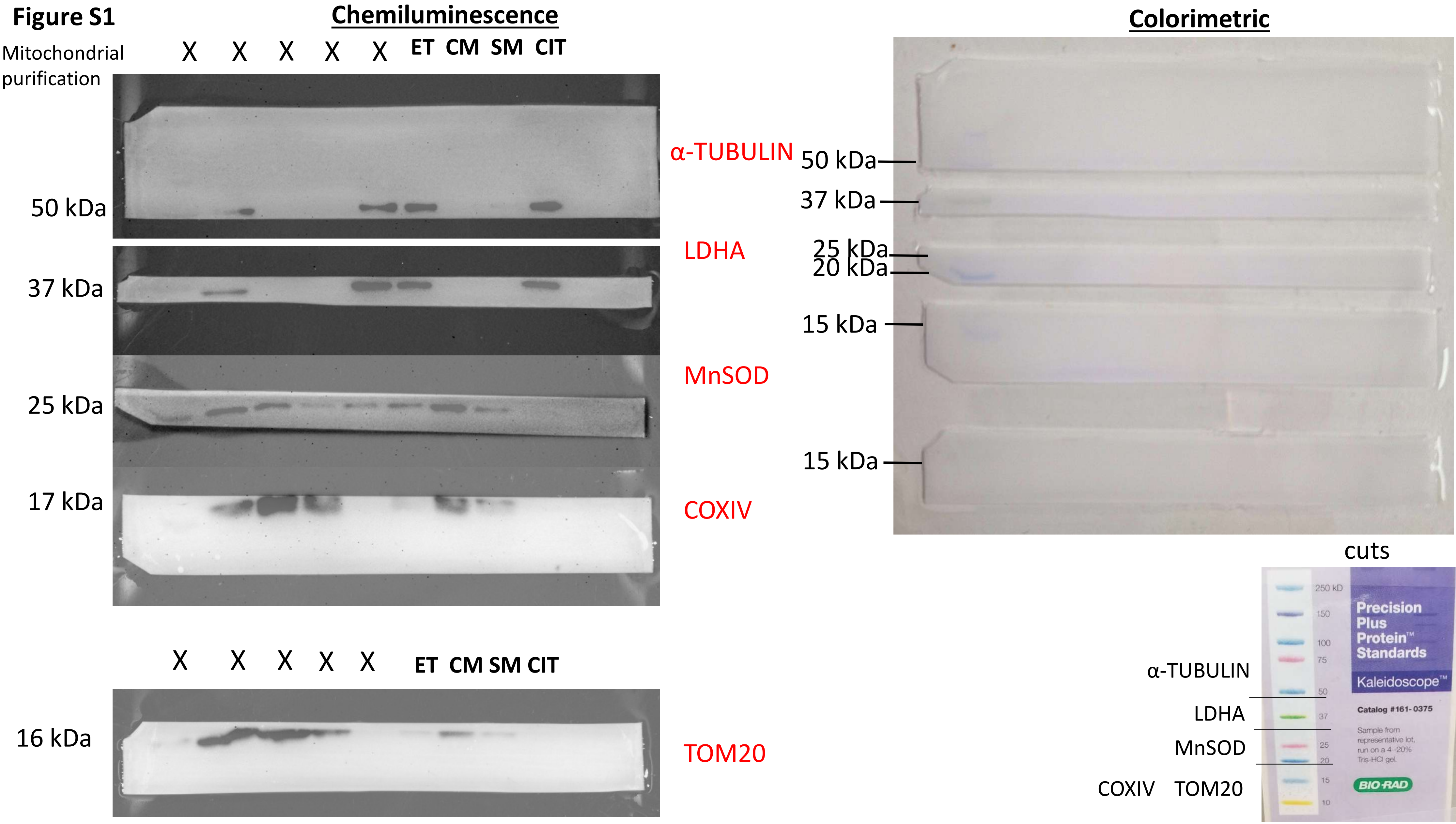
Chemiluminescence
Mitochondrial purification
X X X X X ET CM SM CIT
50 kDa
α-TUBULIN
37 kDa
LDHA
25 kDa
MnSOD
17 kDa
COXIV
X X X X X ET CM SM CIT
16 kDa
TOM20
Colorimetric
50 kDa
37 kDa
25 kDa
20 kDa
15 kDa
15 kDa
cuts
α-TUBULIN
LDHA
MnSOD
COXIV TOM20
250 kD
150
100
75
50
37
25
20
15
10
Precision Plus Protein™ Standards
Kaleidoscope™
Catalog #161-0375
Sample from representative lot, run on a 4–20% Tris-HCl gel.
BIO-RAD

Figure S1

Replicate 1

Chemiluminescence

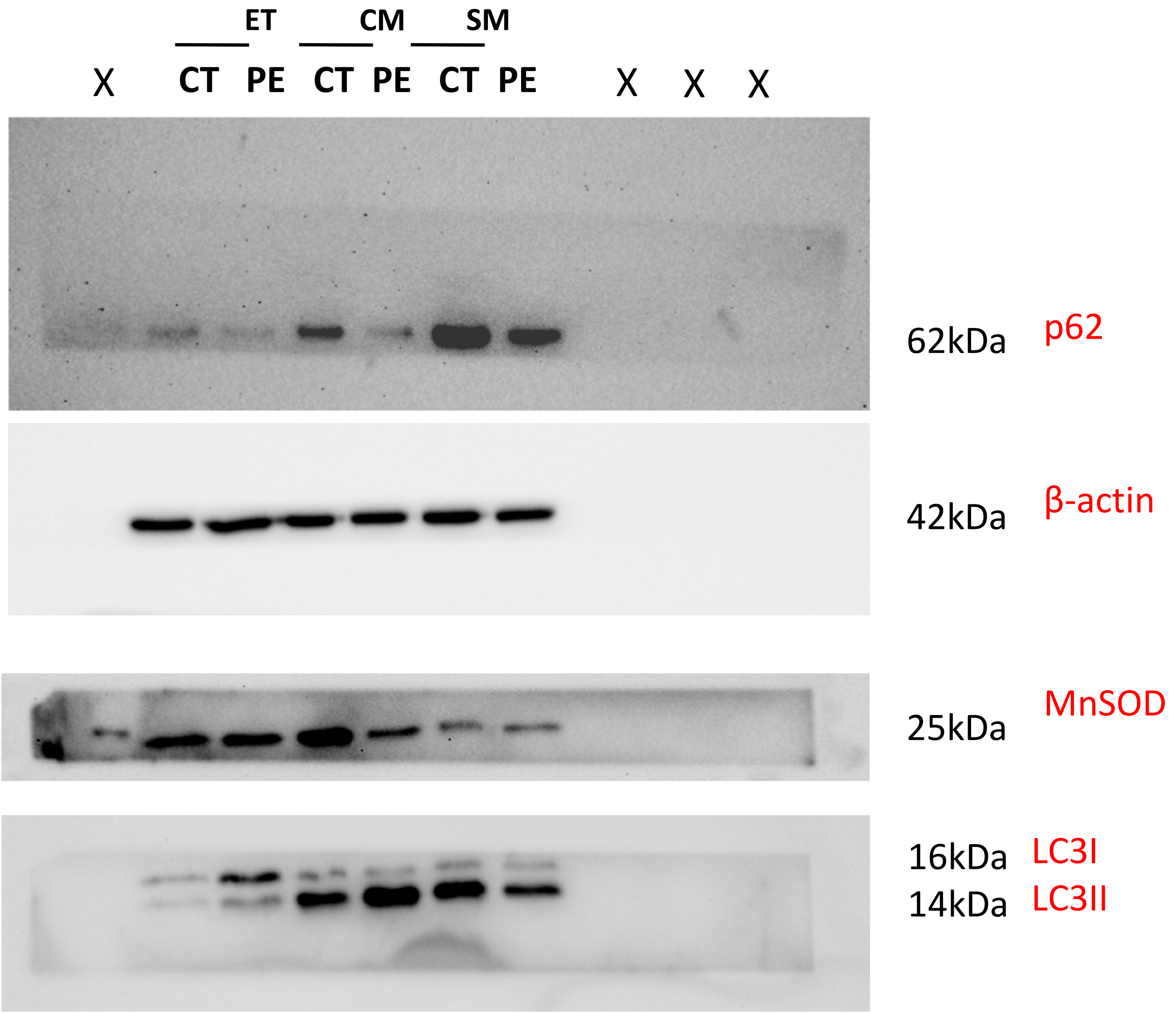

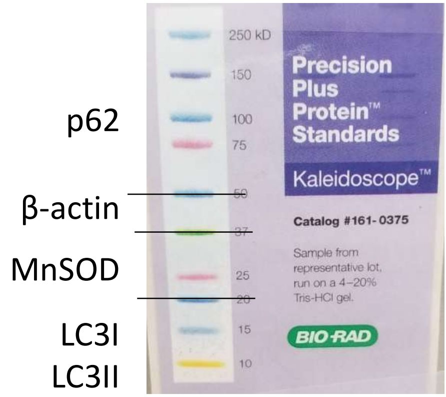

cuts

Colorimetric

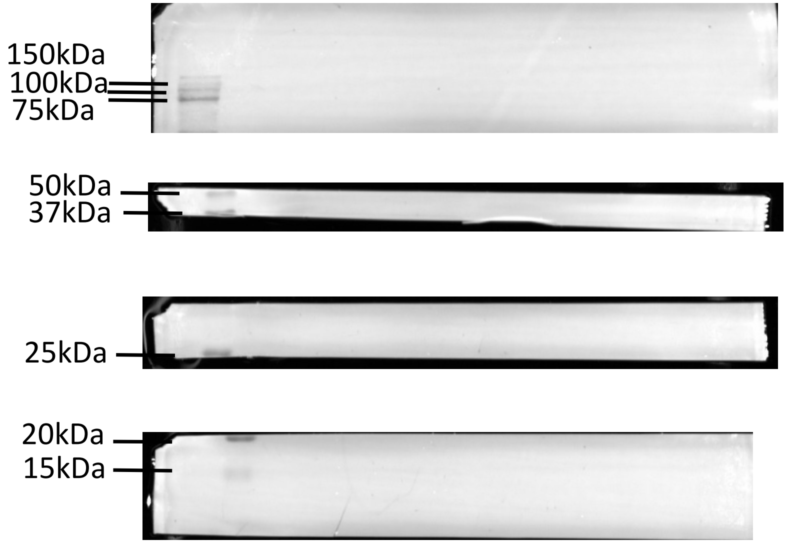

Figure S1
Replicate 2

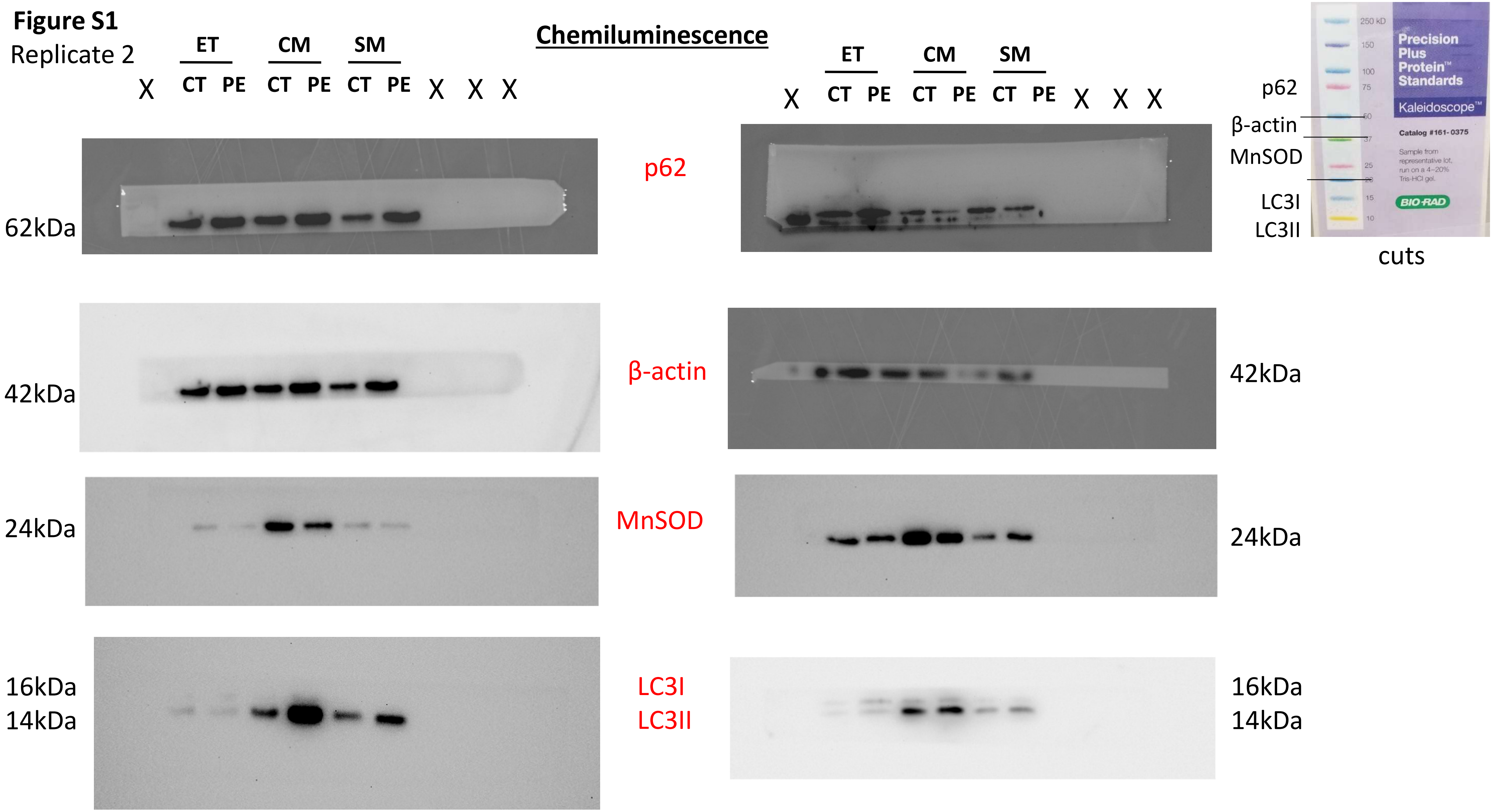

Some membranes are a merge of colorimetric and chemiluminescence.

Figure S1
Replicate 3

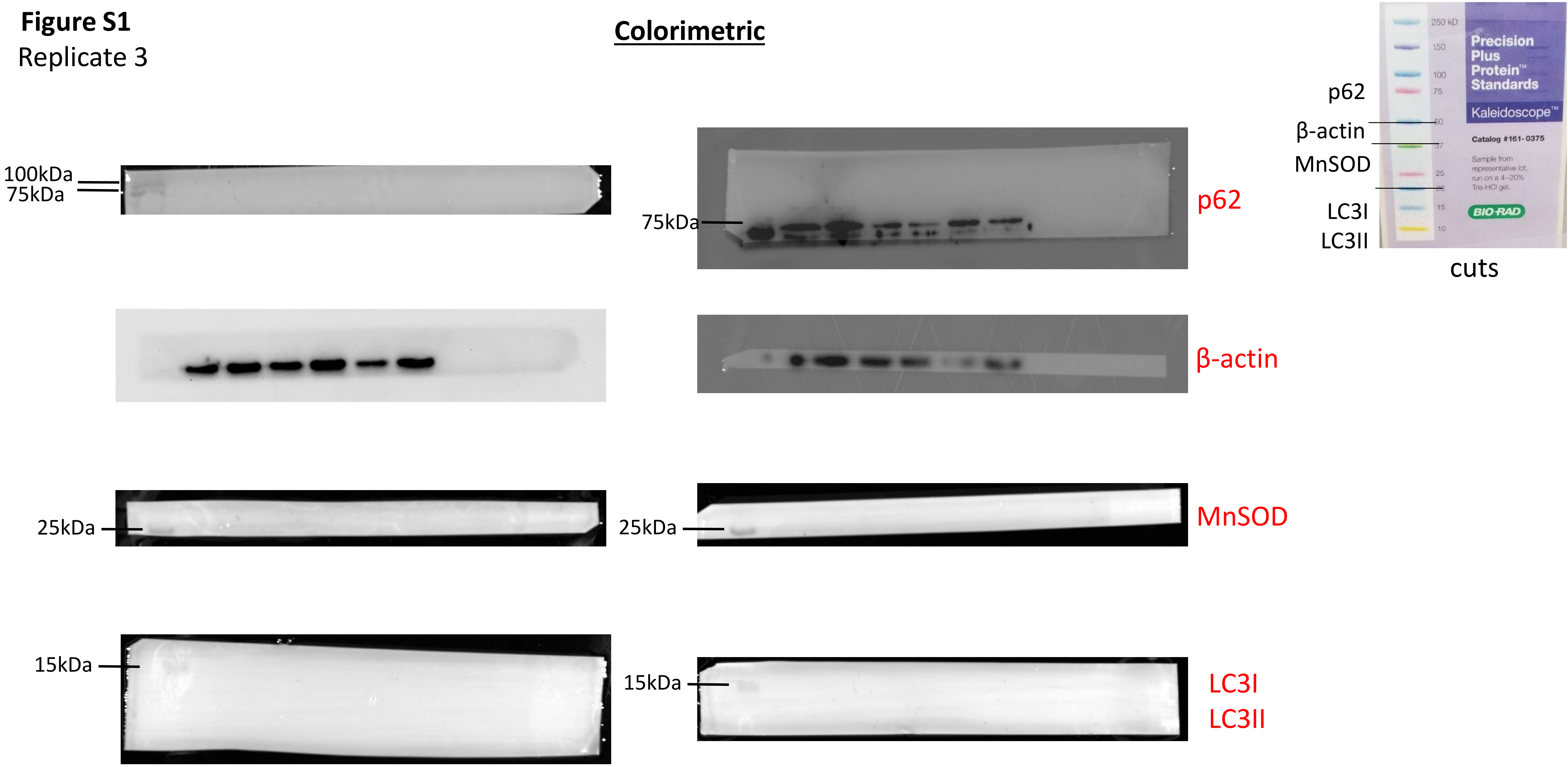

Some membranes are a merge of colorimetric and chemiluminescence.
Due to the cuts, no markers are observed in β-actin

Figure S2 CoQ Chromatograms

A CT Placental sample

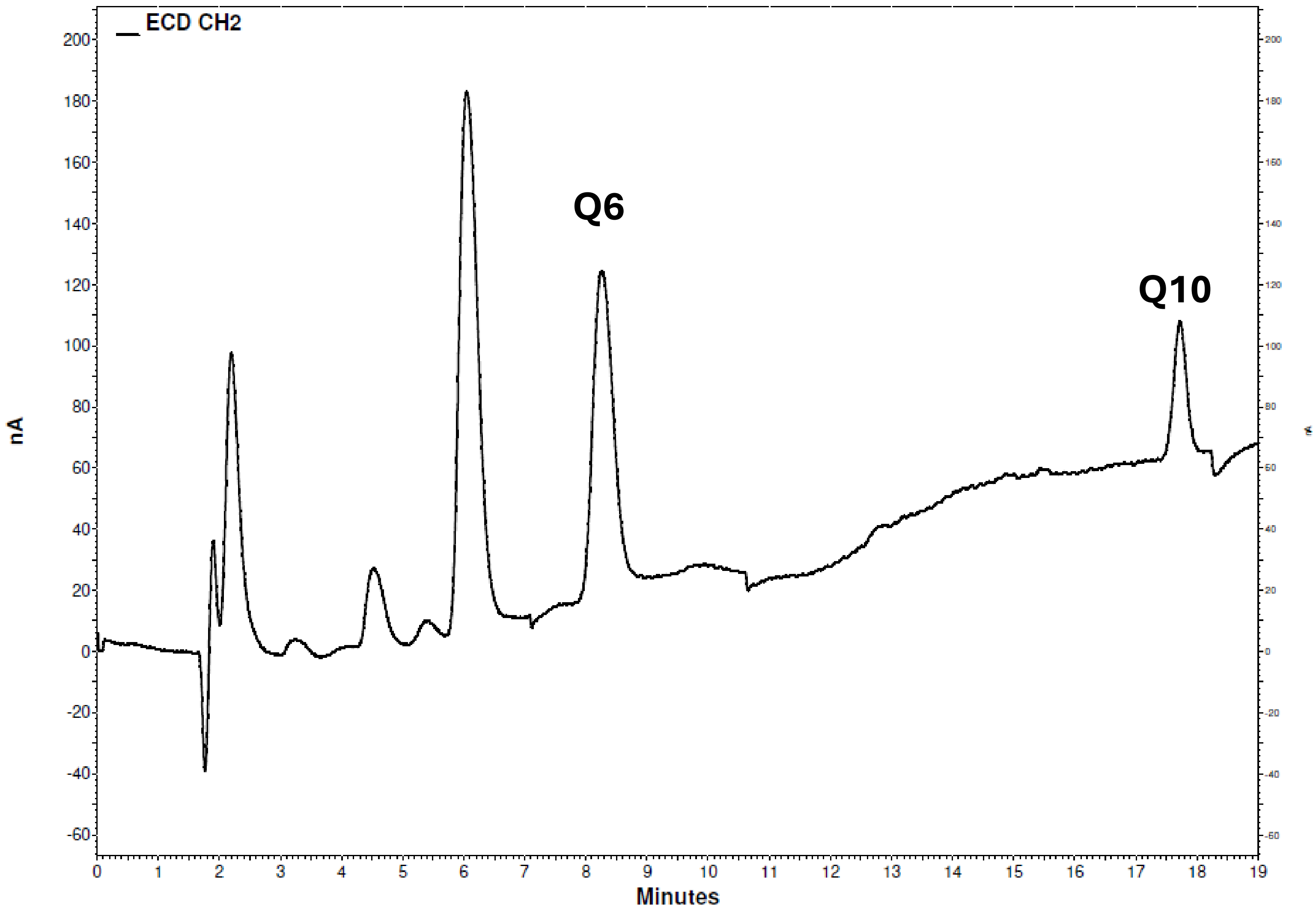

Figure S2 CoQ Chromatograms

B PE Placental sample

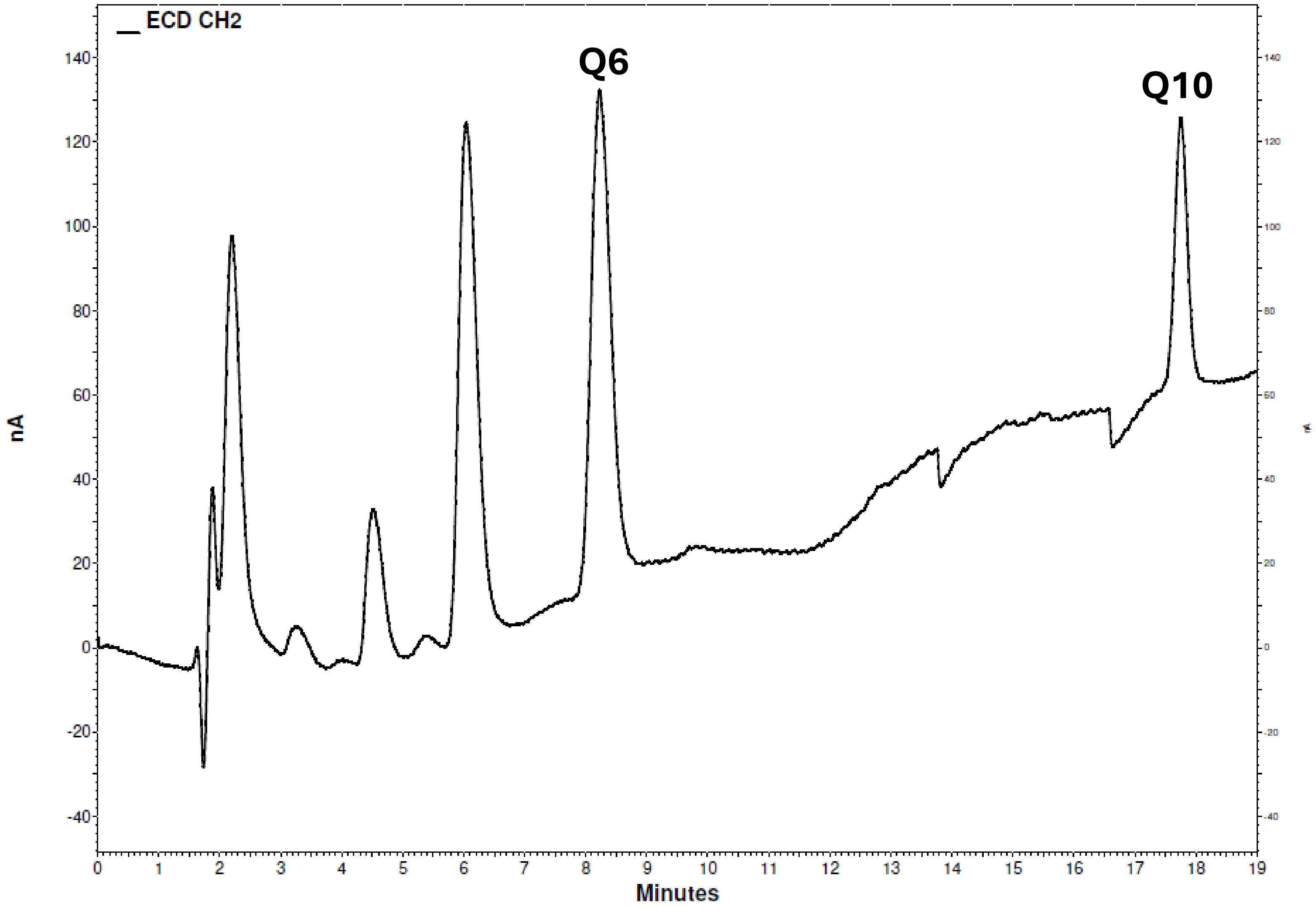

Figure S2 CoQ Chromatograms

C CT CM sample

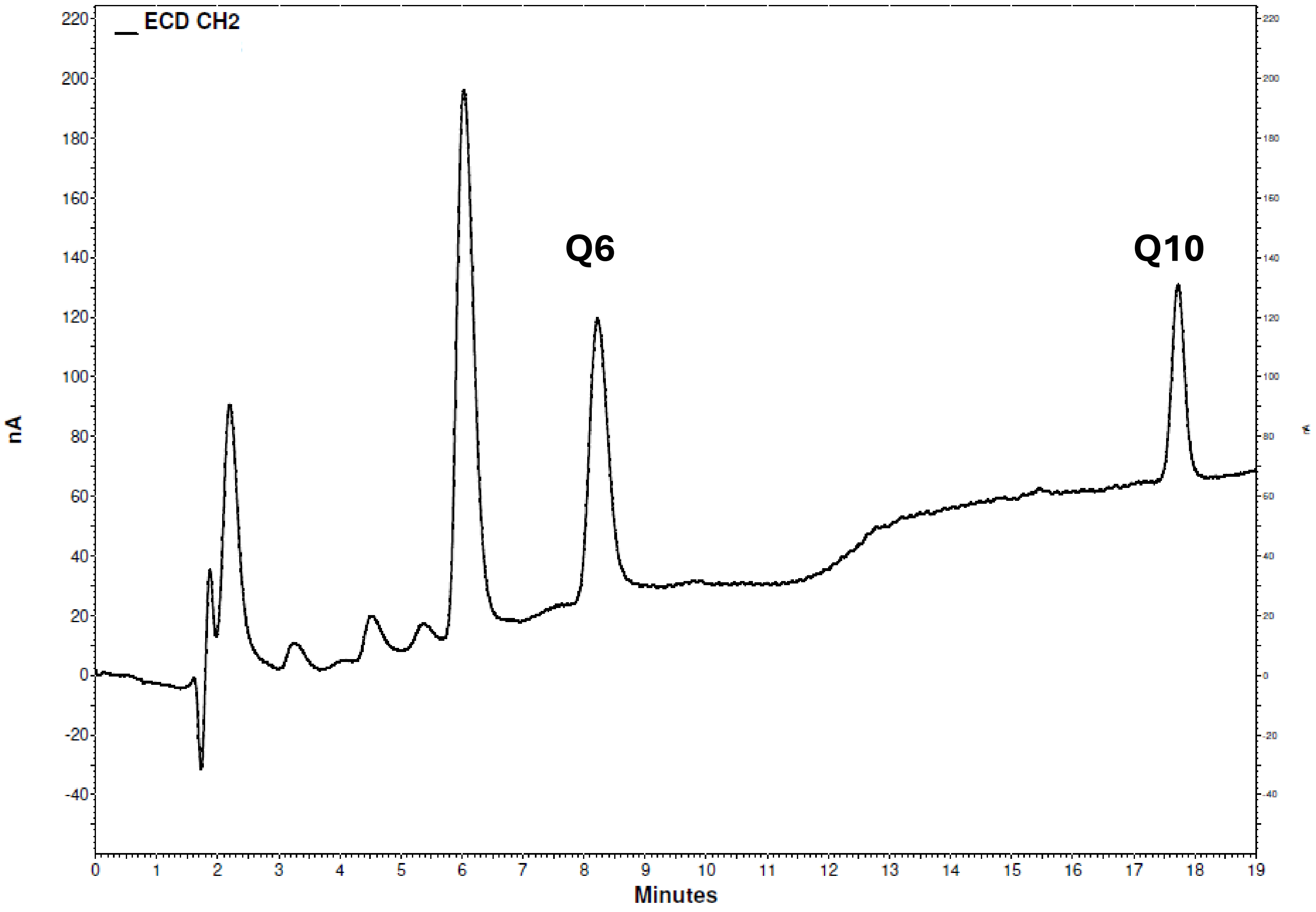

Figure S2 CoQ Chromatograms

D PE CM sample

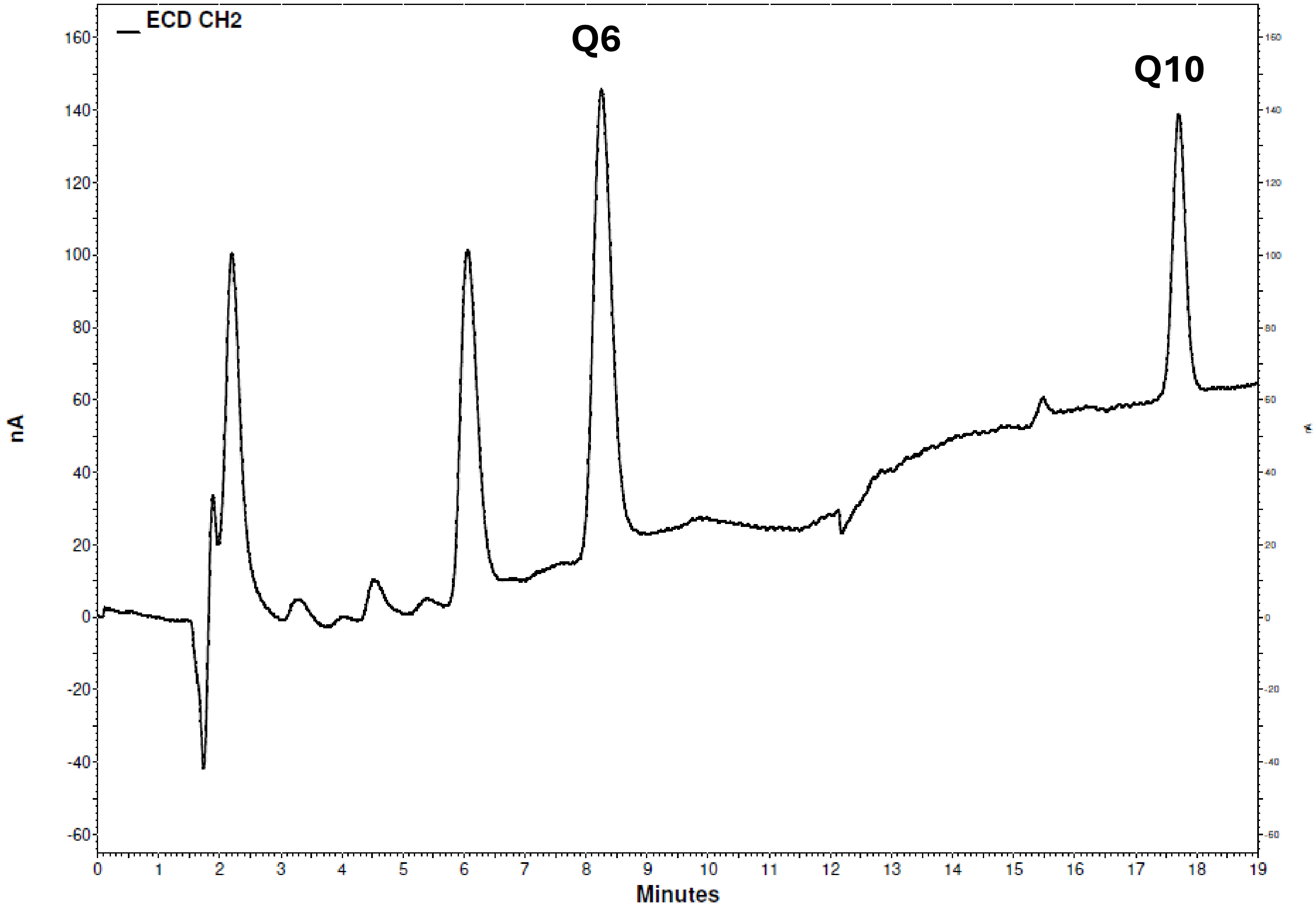

Figure S2 CoQ Chromatograms

E CT SM sample

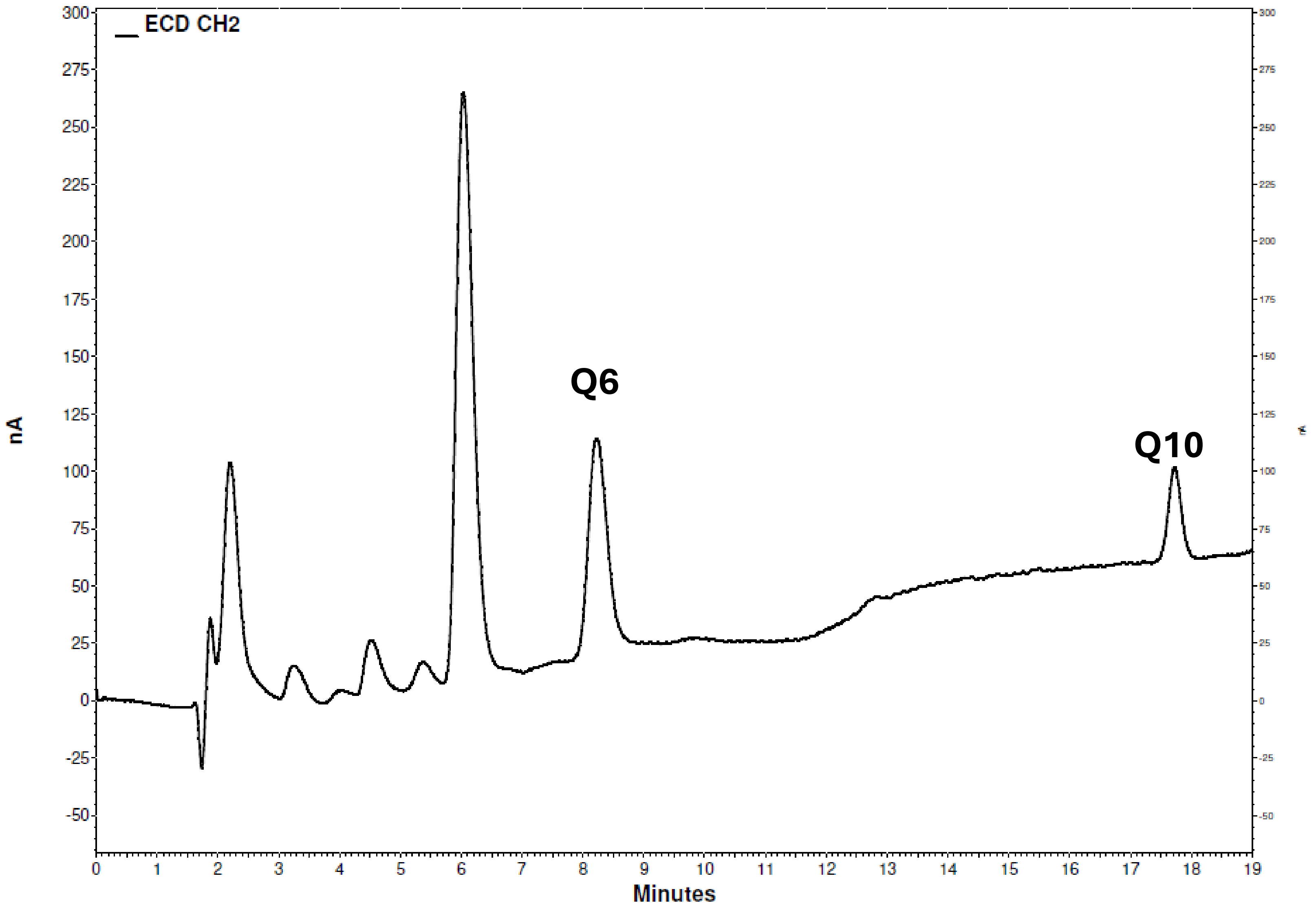

Figure S2 CoQ Chromatograms

F PE SM sample

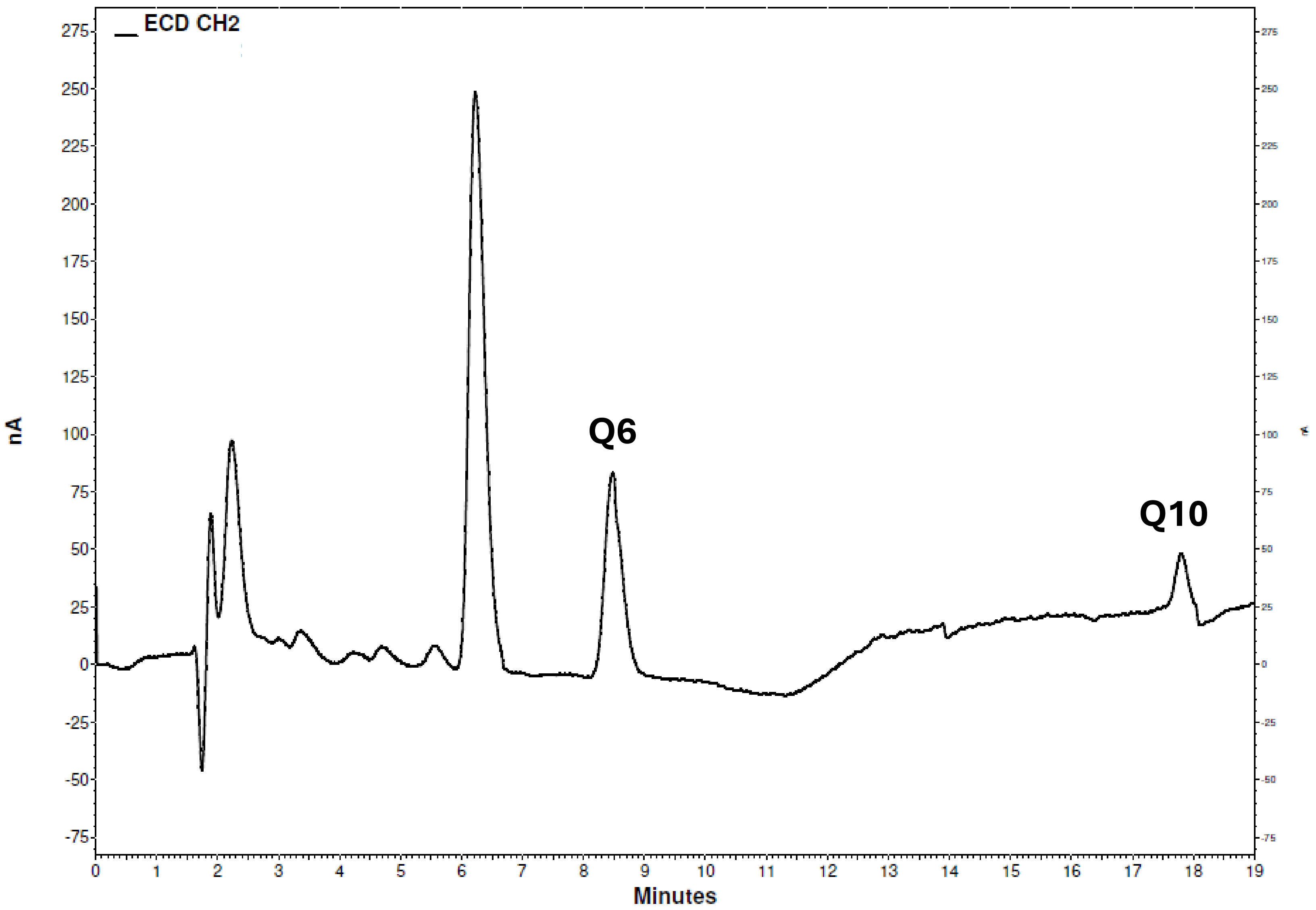

Supplement: Supplementary file 1 [file biology-15-00139-s001.zip › biology-4017557-supplementary.pdf]
